# Holy grail or convenient excuse? Stakeholder perspectives on the role of health system strengthening evaluation in global health resource allocation

**DOI:** 10.1186/s12992-024-01080-6

**Published:** 2024-10-24

**Authors:** Veena Sriram, Natasha Palmer, Shreya Pereira, Sara Bennett

**Affiliations:** 1 School of Public Policy and Global Affairs, School of Population and Public Health, University of British Columbia Vancouver, Vancouver, BC Canada; 2 Queen Margaret University, Musselburgh, UK; 3 Department of International Health, Johns Hopkins Bloomberg School of Public Health, Baltimore, MD USA

**Keywords:** Health systems strengthening, Evaluation, Global health governance, Politics of evidence, Stakeholder analysis, Resource allocation

## Abstract

**Background:**

The role of evaluation evidence in guiding health systems strengthening (HSS) investments at the global-level remains contested. A lack of rigorous impact evaluations is viewed by some as an obstacle to scaling resources. However, others suggest that power dynamics and knowledge hierarchies continue to shape perceptions of rigor and acceptability in HSS evaluations. This debate has had major implications on HSS resource allocation in global-level funding decisions. Yet, few studies have examined the relationship between HSS evaluation evidence and prioritization of HSS. In this paper, we explore the perspectives of key global health stakeholders, specifically around the nature of evidence sought regarding HSS and its potential impact on prioritization, the challenges in securing such evidence, and the drivers of intra- and inter-organizational divergences. We conducted a stakeholder analysis, drawing on 25 interviews with senior representatives of major global health organizations, and utilized inductive approaches to data analysis to develop themes.

**Results:**

Our analysis suggests an intractable challenge at the heart of the relationship between HSS evaluations and prioritization. A lack of evidence was used as a reason for limited investments by some respondents, citing their belief that HSS was an unproven and potentially risky investment which is driven by the philosophy of HSS advocates rather than evidence. The same respondents also noted that the ‘holy grail’ of evaluation evidence that they sought would be rigorous studies that assess the impact of investments on health outcomes and financial accountability, and believed that methodological innovations to deliver this have not occurred. Conversely, others held HSS as a cross-cutting principle across global health investment decisions, and felt that the type of evidence sought by some funders is unachievable and not necessary – an ‘elusive quest’ – given methodological challenges in establishing causality and attribution. In their view, evidence would not change perspectives in favor of HSS investments, and evidence gaps were used as a ‘convenient excuse’. Respondents raised additional concerns regarding the design, dissemination and translation of HSS evaluation evidence.

**Conclusions:**

Ongoing debates about the need for stronger evidence on HSS are often conducted at cross-purposes. Acknowledging and navigating these differing perspectives on HSS evaluation may help break the gridlock and find a more productive way forward.

## Introduction

Health systems strengthening (HSS), defined by the World Health Organization as “any array of initiatives that improves one or more of the functions of the health system and that leads to better health through improvements in access, coverage, quality or efficiency” [1] is widely understood to be key to improving primary health care, achieving universal health coverage and responding to health emergencies [2]. Yet until recently, HSS programs and policies have received limited political priority from national and global level actors, including government and donors [3]. In recent decades, global health donors have directed more attention and financial resources towards HSS [4], although investments remain limited when compared with disease-specific programs [5]. In addition, confusion persists around the exact definition(s) of the term HSS, and in particular whether the aim of HSS investments is structural improvements to the health system or merely further support to shore up short term service delivery aims [6, 7]. Nonetheless, these initiatives have in turn accelerated efforts to evaluate HSS policies and programs and to understand their impact [8], drawing on a diverse set of methodologies and tools from a range of academic disciplines.

Specific challenges have been identified in designing, implementing and utilizing HSS evaluations. First, a common definition for HSS, as well as a framework for HSS evaluations, remains elusive [8–10], complicating efforts to strengthen, coordinate and amplify HSS investments. An illustration of this challenge was described in a study of monitoring and evaluation initiatives pertaining to USAID programs, where the authors found that country missions struggled with identifying appropriate and targeted HSS indicators from a vast pool of potential indicators [11]. Second, donors often wish to attribute impacts on health outcomes to HSS investments; a challenging proposition given the methodological and logical complexity of linking systems-level investments to specific health outcomes [11–13]. Third, evaluative processes and the uptake of evaluation evidence must be viewed through a political lens, with decisions regarding which programs and policies to evaluate, the ownership of evaluation processes, and the systematic utilization of HSS evaluation evidence varying considerably across country contexts [13].

These concerns notwithstanding, the lack of robust evaluation evidence on HSS has remained a key point of concern from some global health funders. This is not surprising: scholarship on priority-setting in global health has highlighted the importance of evidence as one of several factors shaping policy processes that determine commitment and investments from funders [14, 15]. Evaluations of policy and program interventions – a particular form of evidence – have also been identified as a key driver of political priority [16]. For example, the scale-up of performance-based financing in several LMICs can be linked back to promising evaluations from Cambodia and Rwanda [17].

A fundamental tension, however, remains at play with HSS evaluation. While donors continue to seek evaluation evidence to justify both scale up and targeting of HSS investments, the question of what constitutes valid, “acceptable” evidence from HSS evaluations, and importantly, how this evidence is then used for prioritization and decision-making, remains contested [18]. The political nature of evidence use in decision-making has been a key focus in critical analyses of global health policy [19], specifically, the ways in which power dynamics and knowledge hierarchies shape what is perceived as rigor in scientific assessments of program and policy impact [20]. Unlike vertical interventions (such as rolling out a new vaccine), HSS investments do not lend themselves easily to particular evaluation methods, such as randomized control trials (RCTs) [21]. For political reasons, HSS programs and policies often address an entire geography (preventing randomization), and with multiple interrelated elements that interact with the context, it can be extremely challenging to standardize them. HSS investments are also highly varied in nature, meaning that the evaluations cover a wide range of questions and associated methods, generating debate regarding rigor of these approaches and their suitability as an input into priority setting.

To-date there has been limited exploration of the perspectives of global health actors on the relationship between HSS evaluation evidence and political prioritization of HSS. Addressing this gap is particularly important given the existence of two issues that are in contention in this domain – the desire by global health organizations to base their funding decisions on evidence, and the methodological challenges in collecting the type of evidence apparently sought by funders regarding HSS interventions. Despite the growing salience of HSS in global health targets, including high-level commitment to expanding universal health coverage and widespread systems weaknesses observed during the COVID-19 pandemic, progress in overcoming inertia in debates on HSS evaluation evidence and political prioritization has been lacking.

In this paper, we begin to untangle some of these issues by exploring a series of questions – what types of evidence do global health funders seek with regards to HSS evaluations and why? What are the challenges in securing that type of evidence? What are the underlying drivers of intra- and inter-organizational divergences on these issues? And, are there pathways forward to better coordinate and scale HSS evaluations? The findings presented here draw on 25 in-depth interviews with senior representatives of major global health organizations funding and implementing HSS initiatives. The study was part of a series commissioned by the Health Systems Strengthening Evaluation Collaborative (HSSEC), a network of global and national stakeholders seeking to strengthen the quality of evaluations of HSS investments in LMICs and to improve coordination across stakeholders in this space [22].

This paper is organized as follows: having set out the background motivating the study, we next describe the methodology used, followed by a description of key findings. We conclude with a discussion of how our findings engage with the wider literature, and also offer recommendations for how stakeholders may build on these findings. The argument advanced here is that there is an intractable challenge at the heart of the relationship between HSS evaluations and prioritization, where evidence (or the lack thereof) is used as a reason for limited investments, but that the evidence sought by these same funders is unlikely to be achievable given methodological challenges in establishing causality and attribution. Ultimately, this has created an impasse with key stakeholders holding divergent philosophical or ideological perspectives on the role of HSS in global health, but unable to reconcile these by resorting to the type of evidence that they desire.

### Methodology

This study was a prospective stakeholder analysis, utilizing qualitative methods. Qualitative methods such as interviews are common in stakeholder analyses in order to understand individual and organizational perspectives on particular issues, and to delve into topics such as power, interests and relationships [23, 24].

### Data collection

#### Sampling

We utilized purposive sampling to select our respondents [25]. The first step was to develop a master list of potential respondents working primarily at the global-level from multi-lateral agencies, bi-lateral agencies, philanthropic organizations, civil society organizations and research organizations involved in HSS evaluations at the national level. National health authorities were the focus of a different study within the same project; for this reason, only limited recruitment was done with this constituency. The master list emerged through extensive discussions amongst the research team and suggestions of HSSEC members. We also drew on suggestions from respondents involved in the study, using a snowballing technique. Sampling decisions were taken to ensure diversity across types of stakeholders, types of global health investors and positions within the organization.

#### Interview guide development

Appendices 1–3 contain the interview guides (see Box 1 for sample questions). Guides were developed for the following groups : (1) global health investors; (2) research groups and civil society; (3) implementing country stakeholders (i.e., national health authorities, research organizations). Guides were pilot tested with two respondents and then periodically revised to reflect learnings from the interviews. Given the semi-structured nature of the interview process, flexibility was given to interviewers to probe or raise follow-up questions building from responses given within the interviews.

#### Implementation

Interviews were conducted in pairs using the Microsoft Teams or Zoom platforms. Verbal consent and permission to record was sought from all participants. Verbal consent documents and categories of questions were provided to the respondents in advance of the interview.

We conducted 23 interviews with 25 respondents (two interviews were conducted with jointly two respondents) in 2021–2022, as described in Table 1. Further information regarding organizations or organizational affiliations are not provided, in order to protect respondent identities.

**Table 1:** Stakeholder categories and interview respondents

Stakeholder category	Number of interview respondents
Multilateral agencies	8
Bilateral agencies	7
Philanthropic organizations	6
Other (civil society organizations, research organizations)	4
Total	25

### Data analysis

Audio recordings from interviews were transcribed using otter.ai, and then cleaned and checked. Following a preliminary review of the transcripts, we developed a codebook based on inductive categories and codes. Initial themes were developed by the research team through multiple reviews of transcripts and regular debriefing within the team. To further develop specific themes, a framework approach to qualitative data analysis was utilized to facilitate deeper analysis [7]. Interim findings were shared amongst the research team, and with members of the HSSEC, and feedback was utilized to strengthen and modify existing themes.

## Results

### Divergent understandings of health systems strengthening and its purpose

Respondents widely agreed that there was a growing recognition among global health funders of the importance of health systems in achieving global health goals. This has however not been accompanied by a shared vision regarding interventions, policies and programs that will ultimately contribute to HSS. In their view, the lack of a commonly recognized framework to describe and measure health system investments has resulted in two related challenges: difficulties *within* organizations to describe, track and assess HSS investments, and an inability to effectively compare and coordinate investments *across* organizations.

“…so the very kind of notion of what is health system strengthening I think has become very distorted in [global health initiative]. And what’s counted as health system strengthening is largely support.” IDI10, philanthropic organization.

“HSS is such a big animal that sometimes even understanding HSS is a problem. The definition, the concepts, how do we perceive HSS – by both funders, also countries.” IDI13, research organization.

Related to the definitional challenges with HSS were fundamental differences in how global health investors conceptualized HSS within their framework of global health investments. For some respondents, HSS was a distinct set of investments in support of, or in addition to, disease-oriented program investments (e.g., cold chain investments). For others, HSS was viewed as integral to their funding approach to global health and development, and its purpose was a value or guiding principle, rather than a discrete investment. HSS was viewed by these investors as cross-cutting all investment decisions, including disease-specific goals.

“We really feel the health system strengthening aspect is that top priority,and it’s a prerequisite to reach those disease goals and other health goals in reproductive, maternal child health, etc. You know, we feel that health system strengthening is the prerequisite.” IDI12, bilateral agency.

This sense that HSS was a principle or value grounding policy decisions was however viewed in a pejorative sense by some respondents, who noted that evidence – rather than philosophy – was needed to justify reprioritization or redirection of funds.

“People just come out in their camps of you should do strengthening over support, you shouldn’t invest in the verticals, you should just invest in the system strengthening, and that’s your answer…But that bit is not based really on looking at the evidence that… that’s, you know, the principles of what people believe, as opposed to driven by [evidence]. At least the decision making at a board governance level isn’t often driven by that. It’s just people come from their camps of what they think is the right thing” IDI19, philanthropic organization.

“I think we have very different approaches to health systems across agencies. And there’s a downside to, to just sort of throwing all the money into one big pot. And, and I think, again, [to] actually be able to have that conversation based on data rather than just based on philosophy would be helpful.” IDI14, multilateral agency.

Some respondents discussed the ways in which these ideological debates played out in the decision-making processes on the boards of global health initiatives. The lack of alignment on the prioritization and conceptualization of HSS amongst donors to these initiatives translates into competing demands for evidence on HSS programming undertaken by the initiatives, as well as duplication of program goals and initiatives.

“Yeah, I think there’s a few donors who have interest [in HSS evaluation] and people will say that this is of interest…But what they’re really looking for, they don’t know. That’s why we get a bit confused. Because they all want kind of different things. And it’s because they’re not necessarily coming at it from a Board perspective, they’re usually coming at it from probably a broader perspective. And that’s why it gets complex.” IDI2, multilateral organization.

### Trade-offs in global health investment decisions

The sense of a “tradeoff” in investment decisions was pervasive amongst our respondents. In this framing, funding for global health was finite, and investors need to balance multiple dimensions in their decision-making: impact of investment on health outcomes, time to impact (lives saved in the short versus long term), perceptions around the disease- or health-conditions targeted, and national interests on the part of bilateral donors. The role of evidence was raised by some respondents as important in understanding the consequences of these trade-offs and driving the debate.

“And it’s that, that’s what I would love to see and have, because…it fundamentally gets back …to a trade off with limited resources. And so when you were only given a choice of saving a life now, versus, we are willing to trade off lives now for a stronger health system later 10 years’ time. And that just doesn’t wash in a global decision making community, especially when you have [at risk] communities at the table. And so that’s the bit…the nuance of how do you measure? How do you combine the two of measuring, strengthening and how it has an impact in on the current situation… it’s really understanding where your trade off really is.” IDI19, philanthropic organization.

This reasoning however was questioned by a few respondents, including by one who shared the following perspectives on how the lack of evidence on the impacts of HSS investments is used as an excuse for the lack of prioritization.

“there’s this perception of a tradeoff, right? So I would have to sacrifice results to strengthen health systems. And I think that’s the piece that needs to be broken down where you can say, you don’t have to sacrifice results, and you can strengthen health systems.” IDI5, bilateral agency

“With health systems, it’s very hard to have a direct impact. And so… that’s the problem. And then I think, if they hear pressure to do more on health systems in more cross cutting way, evidence then or the lack thereof, gets used as the crutch for not doing it. Right. I don’t know that if there was robust evidence of the effectiveness of health systems, that it would really change. But I think the lack of robust evidence is a convenient excuse.” IDI5, bilateral agency.

According to most respondents, these divergent viewpoints sometimes exacerbated the debates about how to best position HSS within global health initiatives. For example, one respondent described a type of dissonance, where agencies simultaneously recognized the importance of HSS, but also questioned the extent to which their investments in HSS brought about impact.

“I’m hearing two parallel narratives at the same time. One is a recognition that health systems are as important, you know, health systems are as important if not more important than ever, and that prioritization of health systems has to be, kind of both the individual agencies mandates, and to learn lessons from COVID, has to be at the heart of what we all do. But I’m also hearing a narrative that all the money we put into health systems is just flushed down the drain, it really doesn’t achieve anything” IDI14, multilateral agency.

Importantly, staff members and teams *within* organizations were also engaged in a debate regarding the level of financial and program attention to HSS within organizations’ portfolios. A few respondents shared perceptions of limited funding within their organizations that ultimately set up competition across various types of investments and programming. Speaking of internal debates regarding HSS funding allocations, one respondent noted,

“…we have tried to make the case that if we really care about health systems, we should interrogate the allocation [of funds] to say, hey, maybe what we’re getting is not enough. We will be told, what’s the result?” IDI8, philanthropic organization.

### What types of HSS evaluation evidence is prioritized, and why?

Evaluation evidence was viewed by many respondents as critical to making an investment case around HSS, particularly in terms of the impact of HSS investments on health outcomes.

“I think for health system strengthening people [it’s] really showing how the system itself has improved. But, I think other stakeholders would like to show a more of a through line…they’re saying, look, we have limited resources for immunization. Why don’t we just plow it all into immunization? Why even have this health system strengthening window which we don’t know what it actually yields for immunization?” IDI4, philanthropic organization.

Financial accountability was also raised by some respondents as an underlying reason for demanding impact evaluations for HSS investments. Previous experiences with HSS investments that resulted in misuse of funds might have also reduced the appetite for “blanket” HSS investment (such as in the context of Sector Wide Approaches - SWAps) according to a few respondents. The need to attribute the impacts of HSS investments to specific donors was therefore perceived by a few respondents as important for financial accountability.

“… we need the attribution because of the weakness of those government PFM [public financial management] systems. That’s the ultimate…we’re managing risk.” IDI16, bilateral agency.

“Because we don’t believe you have the financial and programmatic assurance to know that the money is being spent in the right way.” IDI19, philanthropic organization

“…the trouble was this lack of trust that there was a, that these resources would be used “responsibly”” IDI5 multilateral agency.

The respondents in this study shared divergent perspectives on the need for attribution or contribution in understanding the impact of their investments on outcomes and financial accountability. While some clearly sought to assess the contribution that HSS investments had on impact, others felt that seeking attribution or contribution in the HSS space was methodologically challenging and potentially unattainable, due to the multi-dimensional nature of HSS investments.

“and I think at a technical level, they understand that, yeah, having a separate procurement system, a separate information system, whatever separate delivery system for each disease is not a good way to go. But where we need to build the evidence, is to show how a unified information system can deliver the accountability needed for those programs, specific results. Right. And this is kind of the holy grail for health systems is, you know, is this idea of separating ends and means, okay, the ends don’t change and delivering on those results. But the means need to be more efficient from a system wide perspective, but still deliver those results. And so analysis of evidence that can demonstrate that would be really welcome. We have more evidence of problems created, I would say, but less of results.” IDI7, multilateral agency

“…I think some people are on what’s more than just a somewhat elusive quest. Yeah, evidence on what works to strengthen health systems? If someone asked that question, you know, someone’s sitting in… London or DC or Seattle is saying, where’s my evidence on what works in strengthening health systems? I don’t think that’s an answerable question. Because, like, what part of the system are you trying to strengthen? And in what way and what context?” IDI3, multilateral agency.

Respondents in our study representing global health donors diverged considerably in the *nature* of HSS evaluation evidence which they sought, and in the *end purpose* for gathering that evidence. Respondents articulated three broad sorts of evidence as described in Table 2 and below.

**Table 2:** Nature of evidence sought by global health investors

	Nature of Evidence Requested		
	Impact of HSS Investments	Relative effectiveness of HSS investments	Implementation research, policy analysis & other forms of systems research
Rationale	• To demonstrate effectiveness (or lack thereof) of HSS investments in improving health outcomes • To guide decision making between HSS investments versus other (e.g. disease specific) investments	To guide investment decisions within HSS (e.g. whether to target health information systems or health workforce strengthening)	To provide insight on how best to implement HSS strategies.
Methodological considerations	• Need to assess attribution or contribution of HSS to impact • Evaluating complex bundles of HSS interventions that might address different health system building blocks • Cost effectiveness analysis vis a vis other non-HSS investments. • Affected by time frame – HSS investments may require longer time to impact and therefore require longer evaluation timeframes	• Cost effectiveness analysis of different types of HSS interventions • Concerns about ability of health systems to absorb certain types of HSS investments	• Implementation research to address questions of “how” best to implement HSS • Political economy analysis to assess and address resistance to HSS • Approaches to integrating tacit knowledge into the evidence base

### Impact of HSS investments

Many respondents stressed the importance of measurable results and an analysis of impact or return on investment, described variously as direct and measurable improvements in health outcomes or economic returns. For example, evaluation evidence that could provide a “line of sight” between investments, outputs and health outcomes or impacts in terms of “lives saved” that could be tracked within budgeting cycles were seen by some respondents as critical to further scale up and advocate for HSS investments. Economic returns on investments in the health sector were also seen as valuable in making prioritization arguments.

“At [global health initiative] all the time we’re hearing, okay, well, there’s another study that shown for every dollar that we spend on vaccination is a $28 return or a $63 return, there’s no doubt. Going back to that spending on a vaccine, spending on a vaccine is a fantastic investment. But then, do I need to invest also in health workers along with that? And if so, what’s the ratio of return on investments in the health worker? How do I decide when to when to stop investing on the vaccine and start investing more in the healthcare worker?” IDI9, multilateral agency.

Motivations for this type of evidence varied across the organizations sampled. Respondents noted that within global health initiatives, donors to these initiatives were keen to see budgetary requests for HSS based on evidence, similar to the type of discussion around vertical programs’ rate of return. In their view, such evidence was needed to either redirect funds from other programs or increase the overall pool of funds in order to accommodate HSS investments, with an implicit sense that “disease specific” networks had more voice within the system. Bilateral donors expressed the need for such evidence to satisfy lawmakers’ (and by extension, their voters) desire to see results from resources invested.

“…the strength of the disease specific voices is incredibly strong. And there’s no way to counter that voice without the evidence. Where’s the evidence, if we take money from the recommendation of antiretroviral treatment to system strengthening, that it will pay off? And there isn’t any, to be honest.” IDI10, philanthropic organization.

“So it’s a very lengthy process, data driven, rich in kind of evidence, because…it’s like, you want to borrow money from a bank? Well, we have a lot of different projects, it has to be convincing that, yes, that’s worth this amount of money, it’s worth this effort. So data and evidence, it’s, it’s really, really important and we have seen that it’s not enough. I mean, there are not enough those things in health system, unfortunately.” IDI8, philanthropic organization.

Despite a number of global agreements^1^ committing funders to seek to evaluate the contribution of their investments to collective outcomes rather than attributing specific outcomes to their investments alone, some respondents noted that funders were still often interested in attribution, and in some cases, acknowledged the methodological and systemic challenges in securing such evidence. Further, according to one respondent, this focus on attribution rather than contribution was another reason for the lack of uptake of more holistic evaluations.

“But the question that they want to know is by putting their money in [global health initiative], what does that get them for health systems. And there’s not evidence on that…they want attribution. And we can’t do that, that also goes against it’s completely in contradiction of the principles that we say that we want in terms of how we support health system strengthening. So we want to do a contribution approach, bringing together the resources, what to support the countries to do what they want to do, but yet on measurement, they want to know, attribution.” IDI2, multilateral organization.

“So people tended, actually in the past, to look at individual support in HSS, rather than looking at the HSS, holistically, and I think that’s where, the challenges are, because when it comes to the investment, when it’s come to evaluation of HSS, you see, fragmented evaluation….” IDI13 research organization.

### Evidence on relative effectiveness of HSS investments

Some respondents noted that within the domain of HSS programs and policies, evaluation evidence was needed to understand their relative effectiveness in order to guide prioritization. These respondents also indicated that evidence of relative effectiveness, such as comparing cost effectiveness of interventions, were not widely available and required a more robust evidence base.

“For every country, just give me a league table that shows me the relative cost effectiveness of different sorts of health systems strengthening interventions so that I can just say, Okay, well wait a minute, I should be starting with I should start with the HMIS, invest this much before I move down to investing this much in cold chain before I move this… something that really synthesizes and pulls together?” IDI9, multilateral agency.

“I think we need to be careful not to be too prescriptive, because there isn’t a magic bullet for system strengthening. I mean, we’re really struggling….there’s no evidence on the cost effectiveness of system strengthening that we can find… there is a massive gap.” IDI15, bilateral agency.

### Implementation research, policy analysis and other forms of systems research

A few respondents noted the importance of other forms of research – such as implementation research, policy analysis and other types of systems research – as part of HSS evaluations, in order to better understand the pathways to effective HSS. Some respondents who supported this approach noted the challenge of primarily relying on standard evaluation approaches to highly contextualized programs and policies pertaining to HSS, noting that implementation or operational research that delved into the mechanics of HSS programs and policies would provide richer analysis of barriers and facilitators to program success. However, this type of research seemed to be valued by a minority of respondents and was not perceived as having a high priority amongst the majority of global health investors, nor was it believed to move the needle in regard to prioritization HSS as an investment.

“…the economist perspective dominated the evaluation approach to PBF (performance-based financing) in the last seven or eight years. At the expense of what many of us were arguing for, which is more of the operational implementation research at the local level. Now, I’m hoping that because of…the limited utility of some of these RCTs, the wheels will swing back more towards implementation, operational research…” IDI6, multilateral agency.

### Designing, conducting and translating HSS evaluations

Respondents shared a range of viewpoints regarding the suitability of existing methods to evaluate and track HSS investments, with some suggesting that methodological innovation is required. One of the underlying issues, as described by several respondents, was an ongoing debate regarding the utility of applying methods designed to evaluate specific interventions, specifically RCTs, to assess impact of HSS interventions. Drawing on their widespread use in medicine, the health sciences and increasingly economics, RCTs have been perceived by some to provide higher standards of evidence on effectiveness. However, from a methods standpoint, the utility of RCT-style evaluations in HSS was questioned by some of our respondents due to the complexity and contextually specific nature of HSS investments.

“… there have been a few randomized control trials, but then people rightly point out the limitations of RCTs for evaluating a systems intervention. And, and so it’s given rise to a whole lot of paralysis…in terms of what counts.” IDI6, multilateral agency.

“one of the challenges with health systems research is randomized controlled trials with control populations or control areas are very difficult to do, because of the politics, you can’t assign interventions to one jurisdiction and withhold them from another jurisdiction and, and keep political peace. And so you have to have fancy approaches or step wedge or, or plausibility designs and so on.” IDI15, researcher.

Concerns around alignment with funding cycles was also raised. While global health initiatives often work in relatively short three-to-four-year cycles, health system investments are needed over longer periods of time, and may also need to be evaluated over longer periods.

“And part of this may be a little bit of the time dimension of the grants. And I think that creates some bias. So Global Fund [is] working on a three-year cycle. And you want to do system strengthening around like information systems and capacities to analyze and so on, it’s not going to be a three-year program. So, I think that’s really I mean, if they could, even if the grant cycle will stay the same, but they could embed that in a longer-term program of work that allows…I mean, even arguably, a five-year Bank cycle isn’t really enough.” IDI7, multilateral agency.

Finally, concerns were raised by respondents regarding the challenges of interpreting evidence from HSS evaluations and then applying that to decision-making. One respondent even compared HSS evidence interpretation to ‘art appreciation’, and another noted the lack of engagement with governments on evaluation results.

“most of the evidence is highly academic, it’s very, very gray, it’s not easy to read, was it a success or not? What was cost effective? So, it’s more art appreciation.” IDI17, bilateral agency

“The last part of the puzzle is not happening, you know, translating all those research fundings into policies at country level, not only at the global level, but also at country level, that is lacking.” IDI13, research organization.

## Discussion

The importance of HSS in achieving global health goals has gained widespread recognition from governments, civil society organizations, industry and philanthropy in recent years [26], most recently in the context of the COVID-19 pandemic [27]. Yet, concerns have remained about the relative lack of investment in HSS interventions from global health donors, as well as continued challenges in converging on a shared set of HSS definitions and goals within the landscape of global health organizations [13]. A bottleneck consistently cited by some in the global health investor community in terms of moving HSS further up on the global health agenda is the fact that evidence on ‘what works’ in HSS and the potential trade-offs with increased HSS investments has been limited [4]. The implications of these debates have arguably been a reluctance to embrace a more diverse and robust range of HSS programs and policies, despite mounting evidence of the importance of systems to achieving global health goals.

This paper examines the range of perspectives at the heart of this gridlock, that is, what are the areas of disagreements as framed and understood by major global health investors in the types of HSS evaluation evidence sought, the reasons for demanding that evidence and the drivers of these divergent viewpoints. From the findings of this research, we advance the argument that there is a major ideological divide underpinning these debates – with individuals within several major global health organizations viewing HSS as an unproven and potentially risky investment, in contrast to others who see HSS as a principle or value that should cut across all investment. The former group views evidence as the “holy grail” that will unlock an acceleration of HSS investments in global health, and that overcoming methodological obstacles can and must be a priority for evaluation experts. The latter group believes this to be an ”elusive quest” at best and a “convenient excuse” at worst, suggesting that the pinning of a lack of prioritization on evaluation evidence distracts from concerns that truly drive this ideological debate – notably, concerns about a diffusion of global health initiatives’ mandates, potential financial mismanagement and longer term timeframes associated with HSS impact that are misaligned with existing funding cycles. The thorny nature of this debate is highlighted in statements from respondents who indicate that the latter group is coming from a place of principle and not evidence, which is regarded as weak justification in comparison to perceptions of a stronger evidence base delivered by actors focused on specific diseases.

This ideological divide manifested in the type of evidence sought from HSS evaluations. Studies that provided evidence on the measurable impact of investments in terms of health outcomes or economic returns, and that also were able to provide “accountability” to those funding such investments remained strongly desired, a finding reported previously in the literature [12]. In our study, those individuals and organizations that viewed HSS as foundational principles believed however that impact evaluations were unlikely to effectively capture the importance of context in mediating HSS programs and policies, might not be warranted for particular research questions, and were typically challenging and expensive to conduct, echoing debates in the literature regarding the limitations of RCTs as an evaluation approach for public health and HSS programs [28]. Power dynamics in the deployment of these framings around types of evaluation evidence were also observed in the decision-making processes within global health initiative boards. By drawing on financial and normative power some key funders are able to advocate forcefully for either side. Similar trends have been observed with regards to health policy and systems research and global health initiatives [29].

This research also raises questions about the translation and utilization of HSS evaluation evidence. Concerns were raised regarding the emphasis on evidence for global-level decision-making purposes, rather than national health system planning and development. This observation has been echoed by evaluation experts involved with global health initiatives [30] and reflects a longstanding pattern of dissonant interests and priorities between global health donors and partner governments [31]. Presumably, prioritizing the voices of governments, country-level stakeholders and communities in evaluation design and uptake will support HSS programming and investments in-country, while contributing to a global evidence base. Issues noted on the challenges of succinctly interpreting or translating context-specific evaluation evidence focused on policy analysis or implementation have similarly been identified in the broader domain of health policy and systems research [29], suggesting that further efforts are required to address ‘supply’ side concerns (i.e., highlighting policy-relevant findings, developing outputs more easily accessible to decision-makers), as well as demand-side barriers (i.e., embedded research projects, long-term networks of researchers and practitioners).

## Conclusion

Our study has found that ongoing debates about the need for stronger evidence on HSS are often conducted at cross-purposes. Acknowledging these differing perspectives on HSS evaluation may go some way to breaking the gridlock and finding a more productive way forward. Doing so might unlock greater dialogue around how to scale and optimize HSS reforms and interventions, invest in evidence generation to address a diverse range of research questions, and strengthen communication and exchange around HSS evaluation findings. The commitment to developing a shared understanding of HSS terminologies is promising, and would be further strengthened by deliberations on the methodological requirements associated with different types of evidence. While rank order listing of the cost-effectiveness of various HSS approaches versus disease-specific interventions that some respondents asked for is, in our view, an elusive quest, it is also undoubtedly true that the HSS evidence base needs strengthening, and that increased data availability and methodological innovations are slowly enhancing the prospects for rigorous HSS evaluations [28]. Broadening the view of the audience to be served so that evaluations combine process and impact assessments so as to inform local health systems initiatives while at the same time guiding global level decision-making would add real value. Acknowledging that whilst attributing changes in health systems performance to specific HSS investments may be challenging, there is still value in tracking trends in health systems performance over time. Finally, gradually building the evidence base can provide some indication of the relative impact of HSS investments, would at least partially address questions raised by our respondents.

## Data Availability

The datasets generated and/or analysed during the current study are not publicly available due to the need to protect confidentiality of participants. De-identified selections of data are available from the corresponding author on reasonable request.

## Footnotes

1 See for example “The Three Ones in Action” UNAIDS, 2005: https://data.unaids.org/publications/irc-pub06/jc935-3onesinaction_en.pdf and the Lusaka Agenda, 2023: https://share-net-ethiopia.org/resources/the-lusaka-agenda-conclusions-of-the-future-of-global-health-initiatives-process/.

## References

[1] Adam T, Hsu J, de Savigny D, Lavis JN, Røttingen J-A, and Sara Bennett. Evaluating Health systems strengthening interventions in Low-Income and Middle-Income countries: are we asking the right questions? Health Policy Plann. 2012;27(4):iv9–19. 10.1093/heapol/czs086. 10.1093/heapol/czs086 23014156

[2] Ataya N, Aluttis C, Flahault A, Atun R, and Andy Haines. Improving the Assessment and Attribution of Effects of Development Assistance for Health. Lancet. 2014;384(9961):2256–59. 10.1016/S0140-6736(14)60791-1. 24973810 10.1016/S0140-6736(14)60791-1

[3] Chee G, Pielemeier N, Lion A, Connor C. Why differentiating between Health System Support and Health System strengthening is needed. Int J Health Plann Manag. 2013;28(1):85–94. 10.1002/hpm.2122. 10.1002/hpm.2122 PMC3617455 22777839

[4] El Arifeen, Shams J, Grove PM, Hansen JR, Hargreaves HL, Johnson M, Johri, and Esther Saville. Evaluating Global Health initiatives to improve Health Equity. Bull World Health Organ. 2024;102(2):137–39. 10.2471/BLT.23.290531. 38313152 10.2471/BLT.23.290531 PMC10835640

[5] Elebesunu E, Ebuka GI, Oke YA, Adebisi, Ifeanyi McWilliams N. COVID-19 calls for Health systems strengthening in Africa: a case of Nigeria. Int J Health Plann Manag. 2021;36(6):2035–43. 10.1002/hpm.3296. 10.1002/hpm.3296 PMC8426817 34350637

[6] Essue BM, and Lydia Kapiriri. Priority setting for Health System strengthening in low income countries. A qualitative case study illustrating the complexities. Health Syst. 2021;10(3):222–37. 10.1080/20476965.2020.1758596. 10.1080/20476965.2020.1758596 PMC8330724 34377445

[7] Gale NK, Heath G, Cameron E, Rashid S, and Sabi Redwood. Using the Framework Method for the Analysis of Qualitative Data in Multi-disciplinary Health Research. BMC Med Res Methodol. 2013;13(1):117. 10.1186/1471-2288-13-117. 24047204 10.1186/1471-2288-13-117 PMC3848812

[8] Gilson L, Erasmus E, Borghi J, Macha J, Kamuzora P, Mtei G. Using stakeholder analysis to support moves towards universal coverage: lessons from the SHIELD project. Health Policy Plann. 2012;27(Suppl 1):i64–76. 10.1093/heapol/czs007. 10.1093/heapol/czs007 22388502

[9] Hafner T, and Jeremy Shiffman. The emergence of global attention to Health systems strengthening. Health Policy Plann. 2013;28(1):41–50. 10.1093/heapol/czs023. 10.1093/heapol/czs023 22407017

[10] Hatt L, Johns B, Connor C, Meline M, Kukla M, and Kaelan Moat. Impact of Health Systems Strengthening on Health. Health Finance and Governance Project. Bethesda, MD: Abt Associates Inc; 2015.

[11] Institute for Health Metrics and Evaluation. Financing Global Health 2021: Global Health priorities in a time of change. Seattle, WA: Institute for Health Metrics and Evaluation; 2023.

[12] Itad. 2022. Health Systems Strengthening Evaluation Collaborative. https://www.itad.com/project/health-systems-strengthening-evaluation-collaborative/

[13] Kentikelenis A, Ghaffar A, McKee M, Zennaro LD, Stuckler D. Donor Support for Health Policy and Systems Research: barriers to Financing and opportunities for overcoming them. Globalization Health. 2022;18(1):106. 10.1186/s12992-022-00896-4. 36564847 10.1186/s12992-022-00896-4 PMC9782264

[14] Kutzin J, Susan PS. Health Systems Strengthening, Universal Health Coverage, Health Security and Resilience. Bull World Health Organ. 2016;94(1):2–2. 10.2471/BLT.15.165050. 26769987 10.2471/BLT.15.165050 PMC4709803

[15] Littoz-Monnet A, and Juanita Uribe. Methods regimes in global governance: the politics of evidence-making in Global Health. Int Political Sociol. 2023;17(2):olad005. 10.1093/ips/olad005.

[16] Mwisongo A, and Juliet Nabyonga-Orem. Global Health initiatives in Africa – Governance, priorities, Harmonisation and Alignment. BMC Health Serv Res. 2016;16(4):212. 10.1186/s12913-016-1448-9. 27454542 10.1186/s12913-016-1448-9 PMC4959383

[17] Naimoli JF, Sweta Saxena LE, Hatt, Kristina M, Yarrow, Trenton M, White, and Temitayo Ifafore-Calfee. Health System strengthening: prospects and threats for its sustainability on the Global Health Policy Agenda. Health Policy Plann. 2018;33(1):85–98. 10.1093/heapol/czx147. 10.1093/heapol/czx147 29121223

[18] Parkhurst J. The politics of evidence: from evidence-based policy to the good governance of evidence. Routledge Studies in Governance and Public Policy. Routledge; 2017.

[19] Patton M. Qualitative evaluation and research methods. Beverly Hills, CA: Sage; 1990.

[20] Shiffman J, and Yusra Ribhi Shawar. Framing and the Formation of Global Health Priorities. Lancet. 2022;399(10339):1977–90. 10.1016/S0140-6736(22)00584-0. 35594874 10.1016/S0140-6736(22)00584-0

[21] Victora CG, Habicht JP, Bryce J. Evidence-based Public Health: moving Beyond Randomized trials. Am J Public Health. 2004;94:400405. 10.2105/AJPH.94.3.400. 10.2105/ajph.94.3.400 PMC1448265 14998803

[22] Shiffman J, and Stephanie Smith. Generation of Political Priority for Global Health initiatives: a Framework and Case Study of maternal mortality. Lancet (London England). 2007;370(9595):1370–79. 10.1016/S0140-6736(07)61579-7. 17933652 10.1016/S0140-6736(07)61579-7

[23] Smith SL, and Jeremy Shiffman. Setting the Global Health Agenda: the influence of advocates and ideas on Political Priority for maternal and newborn survival. Soc Sci Med. 2016;166(October):86–93. 10.1016/j.socscimed.2016.08.013. 27543685 10.1016/j.socscimed.2016.08.013 PMC5034850

[24] Soeters R, Habineza C, Peter BP. Performance-based financing and Changing the District Health System: experience from Rwanda. Bull World Health Organ. 2006;84(11):884–89. 17143462 PMC2627555

[25] Storeng KT. The GAVI Alliance and the ‘Gates Approach’ to Health System strengthening. Glob Public Health. 2014;9(8):865–79. 10.1080/17441692.2014.940362. 25156323 10.1080/17441692.2014.940362 PMC4166931

[26] Swanson RC, Bongiovanni A, Bradley E, Murugan V, Sundewall J, Betigeri A, Nyonator F, et al. Toward a Consensus on Guiding principles for Health systems strengthening. PLoS Med. 2010;7(12):e1000385. 10.1371/journal.pmed.1000385. 21203584 10.1371/journal.pmed.1000385 PMC3006350

[27] Wisniewski JM, Valerie A, Yeager ML, Diana, and David R. Hotchkiss. Exploring the barriers to Rigorous Monitoring and Evaluation of Health Systems strengthening activities: qualitative evidence from International Development partners. Int J Health Plann Manag. 2016;31(4):e302–11. 10.1002/hpm.2339. 10.1002/hpm.2339 26927839

[28] Varvasovszky Z, Brugha R. 2000. A stakeholder analysis, Health Policy and Planning, 15, issue 3, Pages 338–45, 10.1093/heapol/15.3.338 10.1093/heapol/15.3.338 11012410

[29] Witter S, Palmer N, Balabanova D, et al. Evidence review of what works for Health systems strengthening. Where and When? ReBUILD and ReSYST research consortia for FCDO; 2021.

[30] Witter S, Palmer N, Balabanova D, Mounier-Jack S, Martineau T, Anna Klicpera C, Jensen MP-G, and Lucy Gilson. Health System strengthening—reflections on its meaning, Assessment, and our state of knowledge. Int J Health Plann Manag. 2019;34(4):e1980–89. 10.1002/hpm.2882. 10.1002/hpm.2882 31386232

[31] World Health Organization. n.d. Health Systems Strengthening Glossary. Geneva, Switzerland: World Health Organization. https://www.who.int/docs/default-source/documents/health-systems-strengthening-glossary.pdf

